# Side-Group Effect on Electron Transport of Single Molecular Junctions

**DOI:** 10.3390/mi9050234

**Published:** 2018-05-13

**Authors:** Miao-Ling Huang, Fan Zhang, Chen Wang, Ju-Fang Zheng, Hui-Ling Mao, Hu-Jun Xie, Yong Shao, Xiao-Shun Zhou, Jin-Xuan Liu, Jin-Liang Zhuang

**Affiliations:** 1 Key Laboratory of the Ministry of Education for Advanced Catalysis Materials, College of Chemistry and Life Sciences, Zhejiang Normal University, Jinhua 321004, China; huangmiaoling886@163.com (M.-L.H.); 17857585071@163.com (F.Z.); jfzheng@zjnu.cn (J.-F.Z.); yshao@zjnu.cn (Y.S.); 2 Key Lab for Functional Materials Chemistry of Guizhou Province, School of Chemistry and Materials Science, Guizhou Normal University, Guiyang 550001, China; wangchen941013@163.com (C.W.); maohuiling93@163.com (H.-L.M.); 3 Department of Applied Chemistry, Zhejiang Gongshang University, Hangzhou 310018, China; hujunxie@gmail.com; 4 State Key Laboratory of Fine Chemicals, Institute of Artificial Photosynthesis, Dalian University of Technology, Dalian 116024, China

**Keywords:** STM break junction, single-molecule conductance, twist angle, nitro group, steric hindrance

## Abstract

In this article, we have investigated the influence of the nitro side-group on the single molecular conductance of pyridine-based molecules by scanning tunneling microscopy break junction. Single molecular conductance of 4,4′-bipyridine (BPY), 3-nitro-4-(pyridin-4-yl)pyridine (BPY-N), and 3-nitro-4-(3-nitropyridin-4-yl)pyridine (BPY-2N) were measured by contact with Au electrodes. For the BPY molecular junction, two sets of conductance were found with values around 10^−3.1^ G_0_ (high G) and 10^−3.7^ G_0_ (low G). The addition of nitro side-group(s) onto the pyridine ring resulted in lower conductance of 10^−3.8^ G_0_ for BPY-N and 10^−3.9^ G_0_ for BPY-2N, respectively, which can be attributed to the twist angle of two pyridine rings. Moreover, the steric hindrance of nitro group(s) also affects the contacting configuration of electrode-molecule-electrode. As a consequence, only one set of conductance value was observed for BPY-N and BPY-2N. Our work clearly shows the important role of side-groups on the electron transport of single-molecule junctions.

## 1. Introduction

Single-molecular junctions have received much attention in recent decades, as they can allow for learning the properties of single molecules, especially the electron transport through single molecules [1,2,3,4]. Typically, single-molecular junctions are constructed with electrode, molecule, and electrode through scanning tunneling microscopy break junction (STM-BJ) [5,6,7] or mechanical controlled break junction (MCBJ) [8,9]. In electrode-molecule-electrode junctions, molecular structures, electrode materials, anchoring groups, contacting configurations, and environments play important roles in electron transport of single molecular junctions [2,10,11,12,13]. Numerous studies have been reported on the influence of molecular structure on conductance. For example, conjugated molecules are usually more conductive than those molecules with non-conjugated structure [7,14]; the addition of side group(s) would change the single molecule conductance and, typically, electron-donating substituents raise the energy level of frontier molecular orbitals, while electron-withdrawing substituents reduce the energy level of frontier molecular orbitals [15,16,17]. Moreover, the configuration of the molecular backbone can be changed when the substituent is added on the adjacent two pyridine rings, as a consequence, the single molecular conductance will be changed [6,18,19]. Despite several studies having been reported, there is still much room for better understanding of the role of side groups on single-molecular conductance.

**Figure 1:**
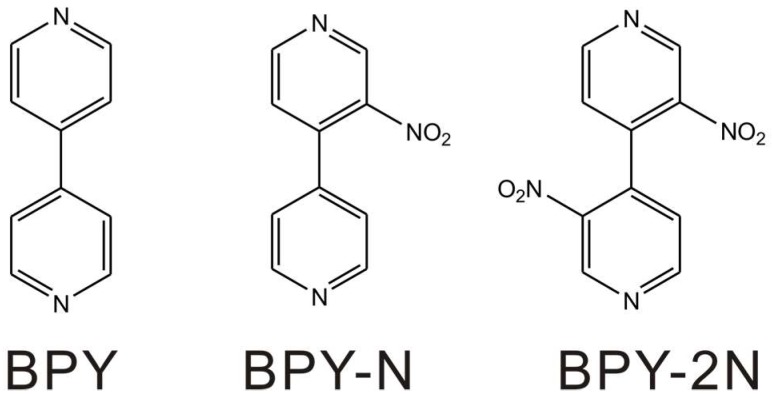
Molecular structures of 4,4′-bipyridine (BPY), 3-nitro-4-(pyridin-4-yl)pyridine (BPY-N), and 3-nitro-4-(3-nitropyridin-4-yl)pyridine (BPY-2N).

Herein, we reported the effect of nitro side-group(s) on the conductance of single-molecular junctions formed with Au electrodes by using the scanning tunneling microscope break junction (STM-BJ) technique. Pyridine-based molecules (Figure 1) were chose to form single-molecular junctions owing to their excellent ability in the form of molecular junctions [5]. Detailed studies reveal that the electron-withdrawing nature of nitro groups, the twist angle of the two pyridine rings, as well as the steric hindrance of nitro group(s) contribute to the electron transport of single-molecule junctions.

## 2. Materials and Methods

4,4′-Bipyridine (BPY) was bought from Alfa Aesar (Ward Hill, MA, USA), while 3-nitro-4-(pyridin-4-yl)pyridine (BPY-N) and 3-nitro-4-(3-nitropyridin-4-yl)pyridine (BPY-2N) were synthesized according to a reported procedure with slight modification [20] (detail of the synthesis can be found in the Supplementary Materials).

Conductance measurement was performed on a Nanoscope IIIa STM (Veeco, Plainview, NY, USA), which was modified to perform the scanning tunneling microscope break junction (STM-BJ) [11,21]. Au(111) and Au wire were used as the substrate and tip, respectively. Prior to each experiment, the substrate was annealed by butane flame. The STM tip was prepared by cutting a 0.25 mm gold wire. The Au(111) was immersed into tetrahydrofuran solution containing 0.1 mM target molecule for 10 min, then washed by tetrahydrofuran solvent. The STM-BJ method is based on the mechanical crash process between tip and substrate. Firstly, the tip was approached to the substrate forming Au atomic contact, then the tip was pulled out from the substrate at withdrawal speed 20 nm/s. During the process, the atomic contact would break, leading to the formation of molecular junctions. The molecular junctions also break during depart of the tip and substrate. Meanwhile, the current of the tip was recorded at a sampling rate of 20 kHz. Thousands of tip current curves were recorded to construct the conductance histogram without data selection. The conductance measurement was carried out at a bias voltage of 100 mV and normalized by the number of used curves.

The twist angle was obtained through theoretical calculation. All compounds were fully optimized by the DFT calculations at the M06 level [22,23,24]. The 6-31G(d,p) basis set [25,26] was used for the C, H, O, and N atoms. Frequency analyses have been carried out to validate the stationary points as minima. All calculations were implemented via the Gaussian09 software package [27].

## 3. Results

**Figure 2:**
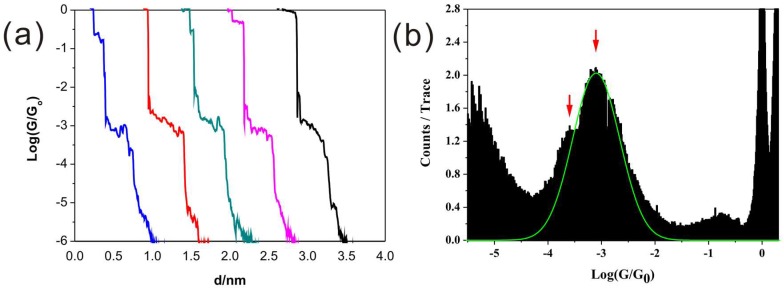
(**a**) Conductance curves and (**b**) conductance histogram of BPY measured at a bias of 100 mV.

Firstly, the single-molecular conductance of 4,4′-bipyridine was measured with Au as the electrode. The conductance curves show stepwise decay around 10^−3.1^ G_0_ (Figure 2a). Meanwhile, conductance histogram constructed from more than 1500 curves shows the pronounced peak at 10^−3.1^ G_0_ (high G) in Figure 2b. This value is comparable with previously reported results [7,28]. A small shoulder peak (around 10^−3.6^ G_0_, low G) was observed in the histogram, which can be attributed to the varied contacting configurations between molecule and electrode [28,29]. It has been proposed that the low conductance value corresponds to the fully-extended BPY molecule between the two electrodes. The high conductance is a result of the BPY binding to the electrode with an angle [29,30].

**Figure 3:**
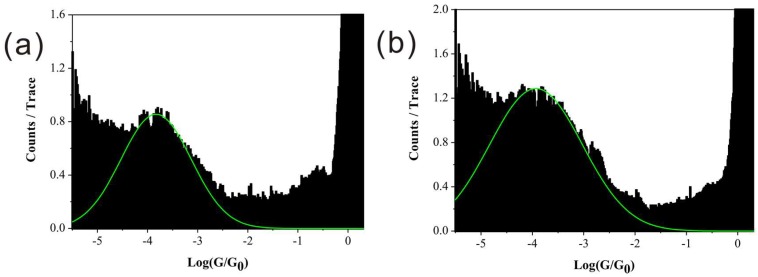
Conductance histograms of (**a**) BPY-N (10^−3.8^ G_0_) and (**b**) BPY-2N (10^−3.9^ G_0_).

Then, we measured the conductance value of BPY-N, consisting of one nitro (-NO_2_) group on BPY. The nitro side-group was demonstrated to influence the electron transport of molecular junctions [15,31,32,33]. As shown in Figure 3a, only one conductance value of 10^−3.8^ G_0_ was found for BPY-N, which is smaller than that of BPY (10^−3.1^ or 10^−3.6^ G_0_). It has been reported that the nitro group could lower the frontier molecular orbital due to its strong electron-withdrawing effect [16,34,35]. For pyridine-based molecule, LUMO dominated electron transport is demonstrated by many works [36]. Thus, the nitro group can increase the single-molecule conductance of BPY-N, which is opposite to our result (G_BPY-N_ < G_BPY_).

Finally, the single-molecular conductance of BPY-2N, bearing two nitro side groups on BPY, was measured. Again, the histogram only shows one kind of conductance in the low G region with a value around 10^−3.9^ G_0_ (Figure 3b). This value is even smaller than that of BPY-N (10^−3.8^ G_0_). These results are opposite to the rule of influence of side groups [16]. Thus, we believed that there must be other factors, which can also contribute to the single-molecule conductance.

**Figure 4:**
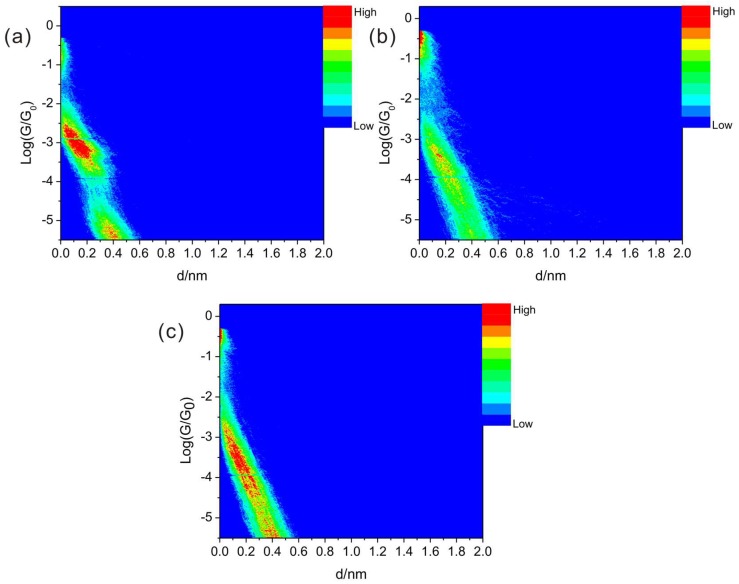
2D conductance histograms of (**a**) BPY, (**b**) BPY-N, and (**c**) BPY-2N. The counts were normalized by the number of used curves for those histograms, and the maximum counts are the same.

The two-dimension (2D) conductance histograms (Figure 4) show similar conductance values as those in one-dimension conductance histograms. Those results show the single conductance values are around 10^−3.1^ G_0_ (with a shoulder peak around 10^−3.6^ G_0_), 10^−3.8^ G_0_, and 10^−3.9^ G_0_ for BPY, BPY-N, and BPY-2N, respectively.

**Figure 5:**
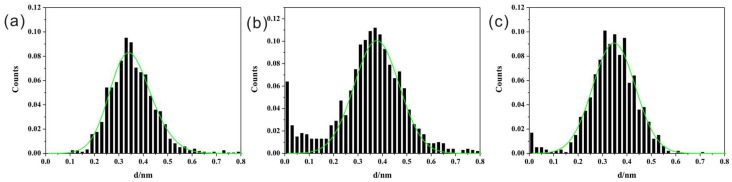
Rupture distance obtained from conductance value of 10^−5.0^ G_0_ to10^−0.3^ G_0_ (0.5 G_0_) in every conductance curve (**a**) BPY, (**b**) BPY-N, and (**c**) BPY-2N.

To gain more information about the single-molecular junctions, we have analyzed the rupture distance of those molecular junctions obtained from conductance values from 10^−5.0^ G_0_ to 10^−0.3^ G_0_ (0.5 G_0_) in every conductance curve. As shown in Figure 5, the rupture distance around 0.35 nm was found for all molecules, indicating that those molecular junctions break at a similar length. By considering the snapback distance (0.50 nm) of the Au electrode upon the breaking of the Au contacts [37], the absolute distance is 0.85 nm, which is comparable with the molecule length (about 0.71 nm) of BPY, as calculated between the center of two N atoms for all molecules. Moreover, the very similar rupture distance values also demonstrate that the BPY molecule, as well as the nitro-group(s) functionalized BPY molecules were mainly connected to the Au electrode by the pyridine-Au configuration, rather than the nitro-Au configuration.

## 4. Discussion

Now we focus on the different sets of conductance values. As stated previously, only the conductance value at low G area was observed for BPY-N and BPY-2N, instead of two sets of conductance values (high G and low G) as shown for BPY. The absence of high G value for BPY-N and BPY-2N might be caused by the steric hindrance of the nitro groups, which prevent the pyridine ring from directly binding to the Au electrode at an angle in the electrode-molecule interface. A similar phenomenon has been reported recently by Ismael and co-workers, they found that the BPY decorated with bulky alkyl side-groups reduces the molecule-electrode coupling strength in the high-conductance geometry, thus preventing the formation of high conductance geometry [38].

Our results give out the conductance values following the order of G_BPY-2N_ < G_BPY-N_ < G_BPY_, the conductance values are decreasing with the increasing of number of nitro groups. Generally, the strong electron-withdrawing nature of nitro substituent lowers the frontier molecular orbitals. As a result, the HOMO-dominated electron transport will be suppressed upon adding nitro groups, leading to decreased single molecular conductance; in contrast, the LUMO-dominated electron transport will be enhanced, resulting in increased single molecular conductance. This effect has been well demonstrated by Xiao et al. [15]. They found that the addition of nitro groups to the oligo(phenylene ethynylene)s (OPE)-based single molecular junctions (anchored with dithiolates to the Au electrodes) significantly decreases the single molecular conductance due to the HOMO-dominated electron transport in the electrochemical surrounding. In contrast, single-molecular junctions formed with pyridine-based molecules are typically LUMO-dominated electron transport; therefore, increased single molecule conductance (upon adding side-group of nitro group) is expected. However, we did not observe this trend in our experiments, suggesting that the influence of nitro side-groups on single-molecule conductance is much more complicated than expected. Recently, Rodriguez-Gonzalez et al. found that the electron-withdrawing groups, such as fluorine side-groups and nitro side-groups, change the orbital of the isolated dithiolated oligophenylene ethynylene molecule (S-OPE) [39], but hardly change the energy gap between Fermi level and the HOMO level of molecule in the junction due to pinning effects [40,41]. They ascribed that the variety of the conductance value caused by the side group might arise from the molecule-electrode interfacial coupling in S-OPE system rather than the electron-withdrawing nature of side groups [39,41]. We believed that, apart from the electron withdrawing effect of the nitro group, the addition of the nitro side-group might also alter the interfacial coupling of pyridine and Au electrodes, thereby causing the unexpected change of single-molecule conductance.

The twist angle between two pyridine rings may be changed upon adding side group. The twist angle has been found extremely important for the single molecular conductance [6,19]. Typically, with the increasing of twist angle between the two rings, the degree of π-conjugation in the molecule decreased, as a consequence, the single molecular conductance decrease. The twist angle was estimated by theoretical calculation as described in the Materials and Methods section. The twist angle for BPY is determined to be 35°, while 52° and 77° are found for BPY-N and BPY-2N, respectively, as listed in Table 1. Following this trend, the single molecular conductance can be G_BPY-2N_ < G_BPY-N_ < G_BPY_, which is consistent with our experimental results. It was reported that the single-molecular conductance is linear with cos^2^*θ*, where *θ* is the twist angle between two conjugated rings [6,18,19]. However, if the low conductance value of BPY was used to plot the conductance vs. cos^2^*θ*, good linear fit is seen in Figure S3. This may further demonstrate that the high-conductance configuration is prevented due to the steric hindrance of the nitro side-group. Furthermore, Mao et al. reported that the molecular structure can influence the contact interaction between anchoring group and electrode [21], such an effect may also exist in the current studied molecules caused by the added nitro group on the pyridine ring. The current work shows the important role of side-groups on the electron transport of single-molecule junctions.

## 5. Conclusions

We have measured the single molecular conductance of pyridine-based molecules bearing nitro-side group(s) using scanning tunneling microscopy. Upon varying the number of nitro side-groups, the values of single molecular conductance follow the order of G_BPY-2N_ < G_BPY-N_ < G_BPY_. The plot between single molecular conductance and cos^2^*θ* shows a good linear fitting, demonstrating that rather than the electron-withdrawing nature of nitro side group, the twist angle between the two pyridine rings is responsible for this unexpected order. Moreover, the steric hindrance of nitro group(s) also affects the contacting configuration of electrode-molecule-electrode. As a consequence, only one set of conductance value was observed for BPY-N and BPY-2N. Our work clearly shows the important role of side-groups on electron transport of single-molecule junctions.

## Figures and Tables

**Table 1:** The twist angle and conductance value of molecular junctions of BPY, BPY-N, and BPY-2N.

Molecule	Twist Angle	Conductance
BPY	35°	10^−3.1^ G_0_, 10^−3.6^ G_0_
BPY-N	52°	10^−3.8^ G_0_
BPY-2N	77°	10^−3.9^ G_0_

## Supplementary Materials

The following are available online at http://www.mdpi.com/2072-666X/9/5/234/s1, Detail of synthesis. Figure S1: ^1^HNMR spectra of 3-nitro-4,4′-bipyridine. Figure S2: ^1^HNMR spectra of 3,3′-dinitro-4,4′-bipyridine; Figure S3: Conductance pyridine-based molecules of BPY, BPY-N and BPY-2N vs. cos^2^*θ*, here *θ* is the twist angle between two rings.

## References

[1] Su T.A., Neupane M., Steigerwald M.L., Venkataraman L., Nuckolls C. (2016). Chemical principles of single-molecule electronics. Nat. Rev. Mater..

[2] Xiang D., Wang X., Jia C., Lee T., Guo X. (2016). Molecular-scale electronics: From concept to function. Chem. Rev..

[3] Kaneko S., Murai D., Marques-Gonzalez S., Nakamura H., Komoto Y., Fujii S., Nishino T., Ikeda K., Tsukagoshi K., Kiguchi M. (2016). Site-selection in single-molecule junction for highly reproducible molecular electronics. J. Am. Chem. Soc..

[4] Isshiki Y., Matsuzawa Y., Fujii S., Kiguchi M. (2018). Investigation on single-molecule junctions based on current-voltage characteristics. Micromachines.

[5] Xu B.Q., Tao N.J. (2003). Measurement of single-molecule resistance by repeated formation of molecular junctions. Science.

[6] Venkataraman L., Klare J.E., Nuckolls C., Hybertsen M.S., Steigerwald M.L. (2006). Dependence of single-molecule junction conductance on molecular conformation. Nature.

[7] Zhou X.S., Chen Z.B., Liu S.H., Jin S., Liu L., Zhang H.M., Xie Z.X., Jiang Y.B., Mao B.W. (2008). Single molecule conductance of dipyridines with conjugated ethene and nonconjugated ethane bridging group. J. Phys. Chem. C.

[8] Yang Y., Chen Z.B., Liu J.Y., Lu M., Yang D.Z., Yang F.Z., Tian Z.Q. (2011). An electrochemically assisted mechanically controllable break junction approach for single molecule junction conductance measurements. Nano Res..

[9] Perrin M.L., Frisenda R., Koole M., Seldenthuis J.S., Gil J.A.C., Valkenier H., Hummelen J.C., Renaud N., Grozema F.C., Thijssen J.M. (2014). Large negative differential conductance in single-molecule break junctions. Nat. Nanotechnol..

[10] Peng L.L., Chen F., Hong Z.W., Zheng J.F., Fillaud L., Yuan Y., Huang M.L., Shao Y., Zhou X.S., Chen J.Z. (2018). Precise tuning of single molecule conductance in an electrochemical environment. Nanoscale.

[11] Chen L., Wang Y.H., He B., Nie H., Hu R., Huang F., Qin A., Zhou X.S., Zhao Z., Tang B.Z. (2015). Multichannel conductance of folded single-molecule wires aided by through-space conjugation. Angew. Chem. Int. Ed..

[12] Zhou X.S., Liu L., Fortgang P., Lefevre A.-S., Serra-Muns A., Raouafi N., Amatore C., Mao B.W., Maisonhaute E., Schollhorn B. (2011). Do molecular conductances correlate with electrochemical rate constants? Experimental insights. J. Am. Chem. Soc..

[13] Arroyo C.R., Leary E., Castellanos-Gómez A.S., Rubio-Bollinger G., González M.T., Agraït N.S. (2011). Influence of binding groups on molecular junction formation. J. Am. Chem. Soc..

[14] Haiss W., Wang C.S., Grace I., Batsanov A.S., Schiffrin D.J., Higgins S.J., Bryce M.R., Lambert C.J., Nichols R.J. (2006). Precision control of single-molecule electrical junctions. Nat. Mater..

[15] Xiao X.Y., Nagahara L.A., Rawlett A.M., Tao N.J. (2005). Electrochemical gate-controlled conductance of single oligo(phenylene ethynylene)s. J. Am. Chem. Soc..

[16] Venkataraman L., Park Y.S., Whalley A.C., Nuckolls C., Hybertsen M.S., Steigerwald M.L. (2007). Electronics and chemistry: Varying single-molecule junction conductance using chemical substituents. Nano Lett..

[17] Kaliginedi V., Moreno-García P., Valkenier H., Hong W., García-Suárez V.M., Buiter P., Otten J.L.H., Hummelen J.C., Lambert C.J., Wandlowski T. (2012). Correlations between molecular structure and single-junction conductance: A case study with oligo(phenylene-ethynylene)-type wires. J. Am. Chem. Soc..

[18] David V., Artem M., Mark E., Markus N., Thomas W., Marcel M. (2009). Chemically controlled conductivity: Torsion-angle dependence in a single-molecule biphenyldithiol junction. Angew. Chem. Int. Ed..

[19] Mishchenko A., Vonlanthen D., Meded V., Burkle M., Li C., Pobelov I.V., Bagrets A., Viljas J.K., Pauly F., Evers F. (2010). Influence of conformation on conductance of biphenyl-dithiol single-molecule contacts. Nano Lett..

[20] Zhang L., Jian Y., Wang J., He C., Li X., Liu T., Duan C. (2012). Post-modification of a mof through a fluorescent-labeling technology for the selective sensing and adsorption of ag+ in aqueous solution. Dalton Trans..

[21] Mao J.C., Peng L.L., Li W.Q., Chen F., Wang H.G., Shao Y., Zhou X.S., Zhao X.Q., Xie H., Niu Z.J. (2017). Influence of molecular structure on contact interaction between thiophene anchoring group and au electrode. J. Phys. Chem. C.

[22] Zhao Y., Truhlar D.G. (2006). Comparative dft study of van der waals complexes:  Rare-gas dimers, alkaline-earth dimers, zinc dimer, and zinc-rare-gas dimers. J. Phys. Chem. A.

[23] Zhao Y., Truhlar D.G. (2006). Density functional for spectroscopy:  No long-range self-interaction error, good performance for rydberg and charge-transfer states, and better performance on average than b3lyp for ground states. J. Phys. Chem. A.

[24] Zhao Y., Truhlar D.G. (2007). The m06 suite of density functionals for main group thermochemistry, thermochemical kinetics, noncovalent interactions, excited states, and transition elements: Two new functionals and systematic testing of four m06-class functionals and 12 other functionals. Theor. Chem. Acc..

[25] Binning R.C., Curtiss L.A. (1990). Compact contracted basis sets for third-row atoms: Ga–kr. J. Comput. Chem..

[26] Gordon M.S. (1980). The isomers of silacyclopropane. Chem. Phys. Lett..

[27] Frisch M.J., Trucks G.W., Schlegel H.B., Scuseria G.E., Robb M.A., Cheeseman J.R., Scalmani G., Barone V., Mennucci B., Petersson G.A. (2009). Gaussian 09.

[28] Chen Z.B., Hong Z.W., Li D.F., Wang Y.H., Zheng J.F., Shao Y., Zhou X.S. (2015). The conductance of pyridine-based molecules measured in ambient air and electrolyte solution: Effect of surrounding. Int. J. Electrochem. Sci..

[29] Quek S.Y., Kamenetska M., Steigerwald M.L., Choi H.J., Louie S.G., Hybertsen M.S., Neaton J.B., Venkataraman L. (2009). Mechanically controlled binary conductance switching of a single-molecule junction. Nat. Nanotechnol..

[30] Kamenetska M., Quek S.Y., Whalley A.C., Steigerwald M.L., Choi H.J., Louie S.G., Nuckolls C., Hybertsen M.S., Neaton J.B., Venkataraman L. (2010). Conductance and geometry of pyridine-linked single-molecule junctions. J. Am. Chem. Soc..

[31] Xu B., Dubi Y. (2015). Negative differential conductance in molecular junctions: An overview of experiment and theory. J. Phys. Condens. Matter.

[32] Gergel-Hackett N., Majumdar N., Martin Z., Swami N., Harriott L.R., Bean J.C., Pattanaik G., Zangari G., Zhu Y., Pu I. (2006). Effects of molecular environments on the electrical switching with memory of nitro-containing opes. J. Vac. Sci. Technol. A.

[33] Cheng J.-F., Zhou L., Wen Z., Yan Q., Han Q., Gao L. (2017). The enhanced spin-polarized transport behaviors through cobalt benzene–porphyrin–benzene molecular junctions: The effect of functional groups. J. Phys. Condens. Matter.

[34] Sun Y.Y., Peng Z.L., Hou R., Liang J.H., Zheng J.F., Zhou X.Y., Zhou X.S., Jin S., Niu Z.J., Mao B.W. (2014). Enhancing electron transport in molecular wires by insertion of a ferrocene center. Phys. Chem. Chem. Phys..

[35] Zotti L.A., Kirchner T., Cuevas J.C., Pauly F., Huhn T., Scheer E., Erbe A. (2010). Revealing the role of anchoring groups in the electrical conduction through single-molecule junctions. Small.

[36] Sun L., Diaz-Fernandez Y.A., Gschneidtner T.A., Westerlund F., Lara-Avila S., Moth-Poulsen K. (2014). Single-molecule electronics: From chemical design to functional devices. Chem. Soc. Rev..

[37] Kaliginedi V.V., Rudnev A., Moreno-Garcia P., Baghernejad M., Huang C., Hong W., Wandlowski T. (2014). Promising anchoring groups for single-molecule conductance measurements. Phys. Chem. Chem. Phys..

[38] Ismael A.K., Wang K., Vezzoli A., Al-Khaykanee M.K., Gallagher H.E., Grace I.M., Lambert C.J., Xu B.Q., Nichols R.J., Higgins S.J. (2017). Side-group-mediated mechanical conductance switching in molecular junctions. Angew. Chem. Int. Ed..

[39] Rodriguez-Gonzalez S., Xie Z., Galangau O., Selvanathan P., Norel L., Van Dyck C., Costuas K., Frisbie C.D., Rigaut S., Cornil J. (2018). Homo level pinning in molecular junctions: Joint theoretical and experimental evidence. J. Phyc. Chem. Lett..

[40] Xie Z., Bâldea I., Smith C.E., Wu Y., Frisbie C.D. (2015). Experimental and theoretical analysis of nanotransport in oligophenylene dithiol junctions as a function of molecular length and contact work function. ACS Nano.

[41] Smith C.E., Xie Z., Baldea I., Frisbie C.D. (2018). Work function and temperature dependence of electron tunneling through an n-type perylene diimide molecular junction with isocyanide surface linkers. Nanoscale.

