# Supplementary material for: Side-Group Effect on Electron Transport of Single Molecular Junctions
Source: Micromachines (Basel). 2018 May 13;9(5):234. doi: 10.3390/mi9050234 (PMC6187264; doi:10.3390/mi9050234)
Supplement: Supplementary file 1 [file micromachines-09-00234-s001.pdf]

micromachines

Supplementary Materials

Side-Group Effect on Electron Transport of Single Molecular Junctions

Miao-Ling Huang [1], Fan Zhang [1], Chen Wang [2], Ju-Fang Zheng [1], Hui-Ling Mao [2], Hu-Jun Xie [3], Yong Shao [1], Xiao-Shun Zhou [1,*], Jin-Xuan Liu [4,*] and Jin-Liang Zhuang [2,*]

[1] Key Laboratory of the Ministry of Education for Advanced Catalysis Materials, College of Chemistry and Life Sciences, Zhejiang Normal University, Jinhua 321004, China; huangmiaoling886@163.com (M.-L.H.); 17857585071@163.com (F.Z.); jfzheng@zjnu.cn (J.-F.Z.); yshao@zjnu.cn (Y.S.)

[2] Key Lab for Functional Materials Chemistry of Guizhou Province, School of Chemistry and Materials Science, Guizhou Normal University, Guiyang 550001, China; wangchen941013@163.com (C.W.); maohuiling93@163.com (H.-L.M.)

[3] Department of Applied Chemistry, Zhejiang Gongshang University, Hangzhou 310018, China, hujunxie@gmail.com

[4] State Key Laboratory of Fine Chemicals, Institute of Artificial Photosynthesis, Dalian University of Technology, Dalian 116024, China

* Correspondence: xszhou@zjnu.edu.cn (X.-S.Z.); jlzhuang@xmu.edu.cn (J.-L.Z.); jinxuan.liu@dlut.edu.cn (J.-X.L.).

1. Detail of Synthesis

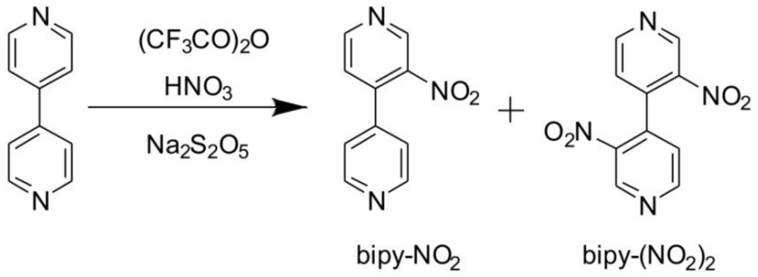

Synthesis of 3-nitro-4,4′-bipyridine and 3,3′-dinitro-4,4′-bipyridine:

The bipy-NO_2 and bipy-$(NO_2)_2$ was synthesized according to a reported procedure with slight modification [1]. Trifluoroacetic anhydride (12 mL) was chilled in an ice bath and 4,4'-bipyridine (3.0 g, 19 mmol) was added in portions and stirred 2 h at chilled conditions. To the chilled suspension, concentrated nitric acid (2.1 mL, 41 mmol) was added dropwise, and the suspension turned into clear solution. After stirring for 12 h at room temperature, thesolution was dripped slowly into a chilled aqueous solution of sodium metabisulfite (3.2 g, 17 mmol in 25 mL of water). After 24 h, the solution was brought to pH 6–7 by addition of 25% NaOH solution. The solution was extracted with DCM (4 x 50 mL), the combined organic phase was dried overanhydrous sodium sulfate; the solvent was evaporated to give the crude product which was furtherpurified by column chromatography using DCM: ethyl acetate (20: 1). First, the 3,3′-dinitro-4,4′-bipyridine was eluted. Elution of 3-nitro-4,4′-bipyridine followed. Evaporation of the solvent resulted in 3,3′-dinitro-4,4′-bipyridine (0.4 g) and 3-nitro-4,4′-bipyridine (1.2 g).

3-nitro-4,4′-bipyridine: 1H NMR: (400 MHz, DMSO-d_6): (δ, ppm) 9.30 (s, 1H). 8.99 (d, 1H), 8.73 (d, 2H), 7.70 (d, 1H), 7.50 (d, 2H). FT-IR (cm^{-1}): 1589 (s), 1533(s), 1519(s), 1408(w), 1384(w), 1358(s), 1190(w), 855(m), 822(m), 765(m), 613(m), 529(m).

3,3′-dinitro-4,4′-bipyridine: 1H NMR: (400MHz, DMSO-d_6): (δ, ppm) 9.5 (s, 2H). 9.0 (d, 2H), 7.7 (d, 2H). FT-IR (cm^{-1}): 1595(s), 1524(s), 1408(w), 1349(s), 1194(s), 855(m), 763(m), 620(m), 589(m).

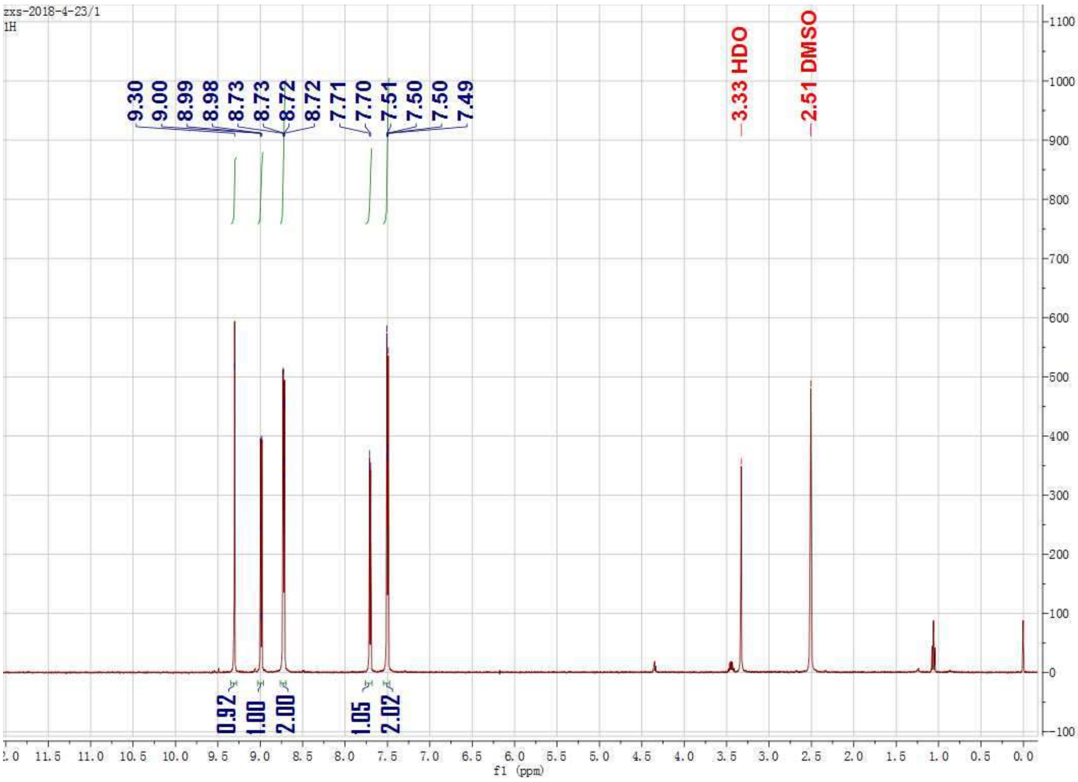

Figure S1. ¹HNMR spectra of 3-nitro-4,4′-bipyridine.

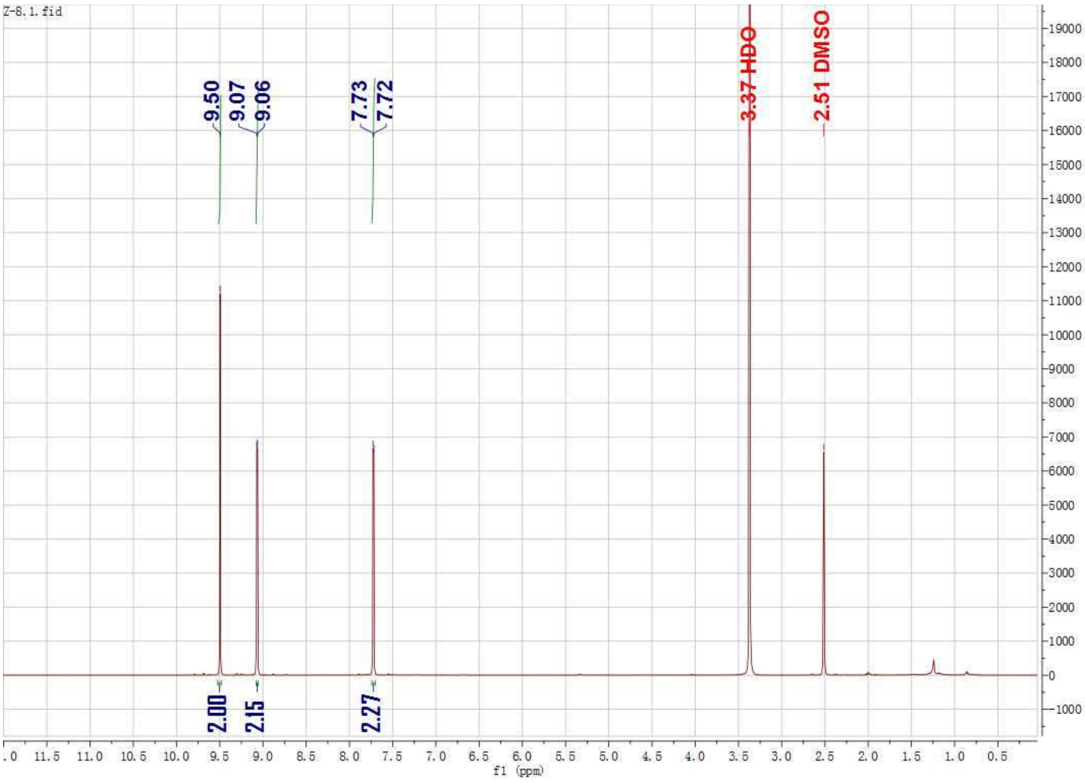

Figure S2. ¹HNMR spectra of 3,3′-dinitro-4,4′-bipyridine.

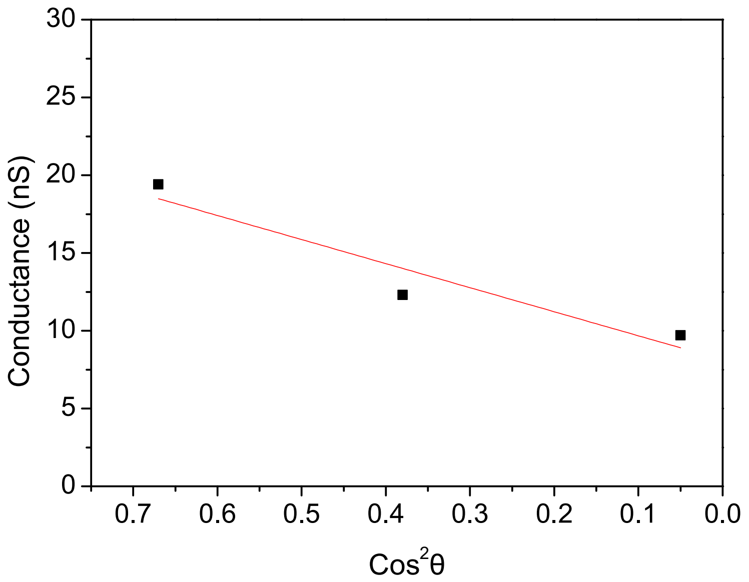

Figure S3. Conductance pyridine-based molecules of BPY, BPY-N and BPY-2N vs. $\cos^2\theta$, here θ is the twist angle between two rings.

Reference

[1] L. Zhang, Y. Jian, J. Wang, C. He, X. Li, T. Liu, C. Duan, *Dalton Trans* **2012**, *41*, 10153–10155.

© 2018 by the authors. Licensee MDPI, Basel, Switzerland. This article is an open access article distributed under the terms and conditions of the Creative Commons Attribution (CC BY) license (http://creativecommons.org/licenses/by/4.0/).
